# Occurrence of West Nile Virus Antibodies in Wild Birds, Horses, and Humans in Poland

**DOI:** 10.1155/2015/234181

**Published:** 2015-03-19

**Authors:** Jowita Samanta Niczyporuk, Elżbieta Samorek-Salamonowicz, Sylvie Lecollinet, Sławomir Andrzej Pancewicz, Wojciech Kozdruń, Hanna Czekaj

**Affiliations:** ^1^Department of Poultry Viral Diseases, National Veterinary Research Institute Pulawy (NVRI), Al. Partyzantow 57, 24-100 Pulawy, Poland; ^2^ANSES, Laboratoire de Santé Animale de Maisons-Alfort, UMR 1161 Virologie, INRA, ANSES, ENVA, 23 avenue du Général de Gaulle, Maisons-Alfort, 94706 Paris, France; ^3^Department of Infectious Diseases and Neuroinfections, Medical University of Bialystok, 14 Żurawia Street, 15-540 Bialystok, Poland

## Abstract

Serum samples of 474 wild birds, 378 horses, and 42 humans with meningitis and lymphocytic meningitis were collected between 2010 and 2014 from different areas of Poland. West Nile virus (WNV) antibodies were detected using competition enzyme linked immunosorbent assays: ELISA-1 ID Screen West Nile Competition, IDvet, ELISA-2 ID Screen West Nile IgM Capture, and ELISA-3 Ingezim West Nile Compac. The antibodies were found in 63 (13.29%) out of 474 wild bird serum samples and in one (0.26%) out of 378 horse serum samples. Fourteen (33.33%) out of 42 sera from patients were positive against WNV antigen and one serum was doubtful. Positive samples obtained in birds were next retested with virus microneutralisation test to confirm positive results and cross-reactions with other antigens of the Japanese encephalitis complex. We suspect that positive serological results in humans, birds, and horses indicate that WNV can be somehow closely related with the ecosystem in Poland.

## 1. Introduction

West Nile virus (WNV) can affect a wide range of bird species, horses, and humans. The virus is an emerging agent responsible for diseases in these animals and humans worldwide. The main vectors of WNV are several species of the blood sucking insects, which can transmit the virus to birds, horses, and humans [1].

The infections are characterised by high pyrexia, paralysis, and morbidity caused by the factors so far acknowledged as pathogenic only for the animals. More often infections are characterised by mild clinical signs [2].

WNV is on the list of the World Organization for Animal Health (OIE) as the neurotropic factor causing the disease under the obligation to notify. West Nile Fever (WNF) caused by the virus is a zoonosis, which is the major public health problem in USA [3].

WNV is an arbovirus belonging to the Flaviviridae family, genus* Flavivirus* included in Japanese encephalitis antigenic complex induced by related antigenically Japanese encephalitis virus (JEV), St. Louis encephalitis virus (SLEV), Murray Valley encephalitis virus (MVEV), and Usutu virus (USUV). The ssRNA+ genome of the virus contains a single open reading frame from 11.000 to 12.000 nucleotides. The genome of the virus consists of seven nonstructural proteins, NS1, NS2a, NS2b, NS3, NS4a, NS4b, and NS5, and three structural proteins: glycoprotein E, core protein C, and premembrane protein prM [4].

Tropical and migratory birds, which belong to different species, are the main reservoir of the virus [5]. Lineage 1 of WNV is a lineage isolated over the world, but lineage 2 was only isolated in Africa and Madagascar, until Hungarian outbreaks of WNV, which was caused by a novel lineage 2, which was introduced to Hungary, most likely by migratory birds from Africa, in 2004 [6]. The same lineage 2 caused in summer of 2010 endemic neuroinvasive disease (WNND) in humans in Greece with 262 confirmed cases and 35 deaths. In 2011 the virus spread to central Greece, but with fewer cases, 101 diagnosed and 9 fatalities. The total number of people in Greece infected by the virus is estimated to 18000 [7].

Over 300 different bird species were infected by the WNV. Usually, the virus circulates between wild birds and mosquitoes in closed cycle and can be carried by the birds during their migrations to the different regions [8].

The virus occurs in many countries around the world and also in 20 European countries, including countries, which are close neighbours of Poland, where the presence of the virus has been confirmed [9–12]. The presence of WNV antibodies was also confirmed in serum samples from wild birds and humans in Poland [13, 14].

In 2010, human morbidity and mortality caused by WNV infection were reported in Greece, Russia, Romania, Italy, and Israel [15]. the circulation of Usutu virus has been also confirmed in Poland [13]. No other studies have been published on the circulation of other viruses responsible for Japanese encephalitis complex in Poland.

The aim of the study was the detection of WNV antibodies in serum samples from different wild birds, horses, and hospitalised patients with neurological symptoms.

## 2. Materials and Methods

**Figure 1:**
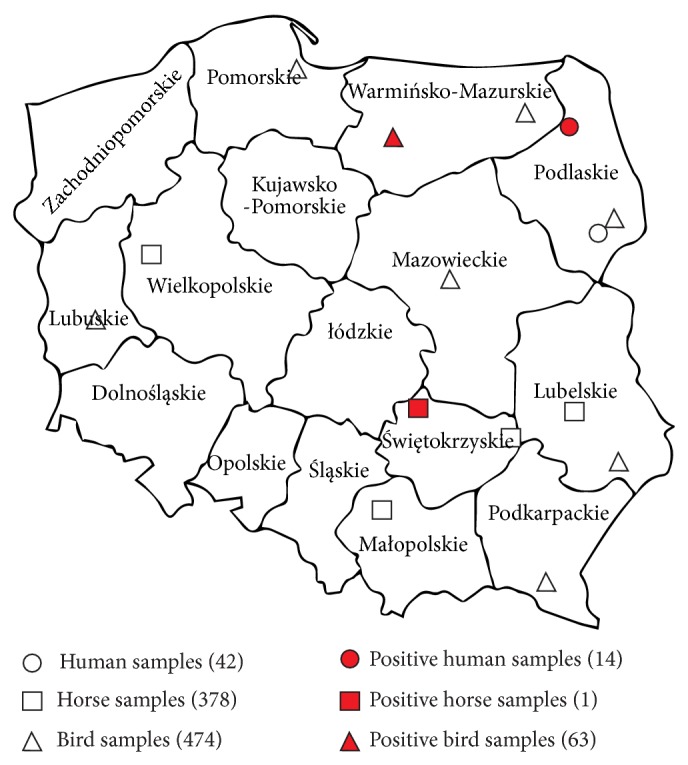
Map of Poland. Areas where the samples were collected.

*Birds.* Serum samples from 474 wild birds, 400 white storks (*Ciconia ciconia*), 21 common pheasants (*Phasianus colchicus*), 19 common chaffinches (*Fringilla coelebs*), nine wild ducks (*Anas platyrhynchos*), four white-tailed Eagles (*Haliaeetus albicilla*), two western capercaillies (*Tetrao urogallus*), six passenger pigeons (*Ectopistes migratorius*), three northern goshawks (*Accipiter gentilis)*, two hooded crows (*Corvus cornix*), three northern goshawks (*Accipiter gentilis)*, and one common swift (*Apus apus*), common blackbird (*Turdus merula*), common starling (*Sturnus vulgaris*), common raven (*Corvus corax*), and common buzzard (*Buteo buteo*), were collected in different locations in Poland (Zoological Gardens, Rehabilitation Centre of Protected Animals). However, most of the samples derived from Wild Birds Rehabilitation Center, Albatros Foundation. Samples have been collected during March–October, 2010–2014, when the mosquito's activity was the highest (Figure 1).

*Horses*. 378 serum samples have been obtained from healthy domesticated horses from few ranches located in four districts. The serum samples were taken also from racehorses (Figure 1). 

*Human*. Serum samples derived from 42 patients (17 men and 25 women) from Department of Infectious Diseases and Neuroinfections, Medical University of Bialystok. The patients displayed neurological symptoms characteristic of meningitis, lymphocytic meningitis, and tick-borne encephalitis. They were hospitalised between May and September, 2010. All patients were tested for tick-borne encephalitis virus antibodies by Enzygnost Anti-TBE/FSME Virus, Siemens [16]. The samples were preserved at −20°C.

All examined samples were double-checked and tested under biosafety level 3+ conditions. 

*ELISA-1.* Serological study was performed with commercially available competition, ELISA ID Screen West Nile Competition (Innovative Diagnostics, Montpelier, France), for detection of West Nile virus antibodies against the pr-E and pr-M envelope proteins containing an epitope common to Japanese encephalitis virus according to the manufacturer's protocol. All samples were examined twice. Microplates were read at 450 nm. The ELISA validated when the residual binding rations (S/N%) were calculated. Serum samples with S/N rations equal to or lower than 40% have been considered as positive; samples with rations higher than 50% were considered as negative. S/N values between 40% and 50% were doubtful.

ELISA-2 was performed using ID Screen West Nile IgM Capture (Innovative Diagnostics, Montpelier, France). Wells were coated with IgM polyclonal antibody. When the positive results were obtained, the presence of antibodies appeared as blue solution and after the addition the stop solution became yellow. In the absence of antibodies, no coloration appeared. Plates were read at 450 nm.

ELISA-3 was performed using Ingezim WNV Compac enzymatic assay (Ingenasa, Spain) based on the blocking ELISA, in which a monoclonal antibody (MAb) specific to the protein E of WNV was used. If antibodies were present in serum samples, they bound to the antigen. When MAb specific to protein E was added, it bound to the antigen that was not blocked by antibodies from serum sample. In case of antibodies blocking the antigen, conjugate did not bind it. The assay was read by a colorimetric reaction after the substrate addition. Samples were considered as positive when the OD values were equal to or lower than the positive control sera samples. Samples were considered as negative when the OD value was equal to or higher than the negative cut-off. Samples with OD value in the range of both values were considered as doubtful.

*Virus Microneutralisation Test*. Virus microneutralisation test with ELISA positive samples from wild birds was conducted in the European Reference Laboratory, ANSES, France. Vero cells and West Nile virus, strain IS-98-ST1, were used in the test. The test was based on the OIE standard procedure [17].

## 3. Results

Districts of Poland and areas where the samples were collected are shown in Figure 1.

Most of the samples were taken from the birds during the assignation and veterinary treatments. Some of the birds were at the rehabilitation center after some accidents. Birds did not manifest any symptoms of neurological diseases and any signs of neurological infections. Every bird was supplied by the organization that send the samples with the information about the location, gender, age, and when it was possible, about travel history.

The examinations of serum samples revealed specific WNV antibodies in 63 samples from wild birds: 62 from white storks and one from common chaffinch. One positive serum sample was obtained from mare.

Concerning human serum samples, 14 out of 42 were positive and one was doubtful. All the patients had history of mosquito and tick bites. Tick-borne encephalitis was diagnosed in 16 patients [16].

To confirm the results, ELISA-2 was performed to detect serum WNV IgM specific antibodies as a first line of immunity response. The results were negative and therefore did not confirm the presence of IgM antibodies or any evidence of recent infection of the animals.

To verify results obtained by ELISA-1, ELISA-3 was performed, and the presence of specific WNV antibodies was confirmed in these cases. The results are presented in Table 1.

For confirmation of positive results, birds' positive serum samples were retested by virus microneutralisation test. All 63 serum samples from wild birds were confirmed and identified as positive for WNV antibodies with the titre 10 in 60 samples and titre 30 in three samples. Concerning examinations of other members of JECV, samples were also negative for Usutu virus. No cross-reactivity reactions were observed.

Comparison of the results obtained by different diagnostic methods (ELISAs and virus microneutralisation tests) is presented in Table 1.

## 4. Discussion

During the last years, it has been noted that WNV spreads in the countries with moderate climate. mosquitoes from the Culicidae family are the most common vector of the virus spreading [8, 10, 18–21] and nowadays six of the mosquito species exist in Polish climate zone [13, 22]. In the nineties of the last century, Juřicová et al. [20] using haemagglutination inhibition assay confirmed WNV antibodies in 12.1% of house sparrows (*Passer domesticus*) and in 2.8% of Eurasian tree sparrows (*Passer montanus*) in Campinas forest area in Poland. In the next few years, WNV antibodies were found in three storks, one crow (*Corvus corone cornix*), and one mute swan (*Cygnus olor*) on the other part of Poland [13].

We have performed three different ELISAs to present and then confirm the obtained results. The first ELISA was ID Screen West Nile Competition, test specific for WNV, but it also gives serological cross-reactions among the JECV members. When positive results were obtained, ELISA-2 West Nile IgM Capture test was performed, which is specific only for WNV and allows excluding cross-reactions but detects only IgM. It means that we can identify only recent infection and cannot detect IgG antibodies. ELISA-3 was used to confirm the obtained results by ELISA-1 and the results were confirmed in 100% of cases.

In our study, positive results with serum samples of wild birds obtained by the ELISAs were confirmed by virus microneutralisation test. Additionally, ELISA showed WNV antibodies in 14 out of 42 serum samples from patients with symptoms of meningitis, lymphocytic meningitis, and tick-borne encephalitis, but we were unable to verify the obtained results with plaque reduction neutralisation test (PRNT). WNV infection in humans and identification of WNV in cerebrospinal fluid were usually confirmed by RT-PCR, Nested PCR [4, 5, 23–26], or in formalin-fixed, paraffin-embedded human tissues by RT-PCR. According to the study of Czupryna et al. [16], all samples of cerebrospinal fluid from 24 patients hospitalised at the Department of Infectious Diseases and Neuroinfections, Medical University of Bialystok, were negative for WNV RNA.

serum samples of humans, birds, and horses were used as proper samples to determine specific WNV antibodies. If we did confirm the specific antibodies in serum samples, the animal or human was considered as having contact with the virus (in Poland or outside the country). It is supposed that positive wild birds could have contact with the virus outside the country. Concerning the positive samples from humans, 11 patients never left the country; that means they had to have the contact with the virus already in Poland. The same is in a case of the horses, which, according to their travel history, never left the country.

On the basis of epidemiological situation in Europe, the role of wild birds in WNV transmission and results of previous serological examinations of humans and birds [10, 20, 22, 26, 27] indicate that the presence of WNV in Poland can be possible. In Poland, WNV antibodies were detected in febrile woman [22] and in healthy forest workers from regions of Swietokrzyskie (28.85%) and Podlaskie (34.14%) [27]. Our serological results can suggest that the virus is already present in our climate zone and in our ecosystem. The possibility of its cross-reaction with viruses of tick-borne encephalitis and endemic disease should be taken into consideration.

## Figures and Tables

**Table 1:** Comparison of results obtained by different methods.

	Serum		
	Wild birds	Horses	Humans
ELISA ID Screen West Nile Competition IDvet	63^*^/474^**^ (13.29%)	1/378 (0.26%)	14/42 (33.33%)
ELISA ID Screen West Nile IgM Capture ID-vet	0/474	0/378	0/42
Ingezim West Nile Compac	63/474	1/378	14/42
Virus microneutralisation test (on positive samples)	63/63	—/—	—/—

^*^Positive sera.

^**^Total number of examined samples.
